# A network perspective on suicidal behavior: Understanding suicidality as a complex system

**DOI:** 10.1111/sltb.12676

**Published:** 2021-02-24

**Authors:** Derek de Beurs, Claudi Bockting, Ad Kerkhof, Floortje Scheepers, Rory O’Connor, Brenda Penninx, Ingrid van de Leemput

**Affiliations:** ^1^ Trimbos Institute (Netherlands Institute of Mental Health) Utrecht The Netherlands; ^2^ Department of Clinical, Neuro and Developmental Psychology Amsterdam Public Health Research Institute Vrije Universiteit Amsterdam Amsterdam The Netherlands; ^3^ Department of Psychiatry Amsterdam University Medical Centers (location AMC) University of Amsterdam Amsterdam The Netherlands; ^4^ Departement of Psychiatry University Medical Center Utrecht Utrecht The Netherlands; ^5^ Suicidal Behaviour Research Laboratory Glasgow University Glasgow UK; ^6^ Department of Psychiatry Amsterdam Public Health Research Institute Vrije Universiteit Amsterdam Amsterdam The Netherlands; ^7^ Department of Aquatic Ecology and Water Quality Management Wageningen University Wageningen The Netherlands

## Abstract

**Background:**

Suicidal behavior is the result of complex interactions between many different factors that change over time. A network perspective may improve our understanding of these complex dynamics. Within the network perspective, psychopathology is considered to be a consequence of symptoms that directly interact with one another in a network structure. To view suicidal behavior as the result of such a complex system is a good starting point to facilitate moving away from traditional linear thinking.

**Objective:**

To review the existing paradigms and theories and their application to suicidal behavior.

**Methods:**

In the first part of this paper, we introduce the relevant concepts within network analysis such as network density and centrality. Where possible, we refer to studies that have applied these concepts within the field of suicide prevention. In the second part, we move one step further, by understanding the network perspective as an initial step toward complex system theory. The latter is a branch of science that models interacting variables in order to understand the dynamics of complex systems, such as tipping points and hysteresis.

**Results:**

Few studies have applied network analysis to study suicidal behavior. The studies that do highlight the complexity of suicidality. Complexity science offers potential useful concepts such as alternative stable states and resilience to study psychopathology and suicidal behavior, as demonstrated within the field of depression. To date, one innovative study has applied concepts from complexity science to better understand suicidal behavior. Complexity science and its application to human behavior are in its infancy, and it requires more collaboration between complexity scientists and behavioral scientists.

**Conclusions:**

Clinicians and scientists are increasingly conceptualizing suicidal behavior as the result of the complex interaction between many different biological, social, and psychological risk and protective factors. Novel statistical techniques such as network analysis can help the field to better understand this complexity. The application of concepts from complexity science to the field of psychopathology and suicide research offers exciting and promising possibilities for our understanding and prevention of suicide.

## 1 INTRODUCTION

**Figure 1:**
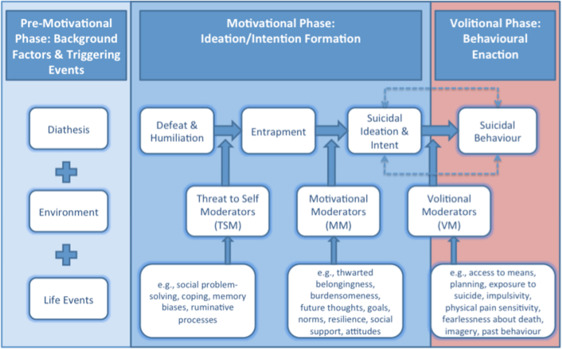
The integrated motivational–volitional (IMV) model of suicidal behavior [Colour figure can be viewed at wileyonlinelibrary.com]

Suicidal behavior poses a major public health problem, with a global estimated 800,000 suicide deaths each year (World Health Organization, 2014). It is estimated that suicide attempts are at least 20 times more common. Different international mental health surveys have found that each year around 3% of the general population experiences a period of at least two weeks in which they have felt that life was not worth living (Nock et al., 2009; Ten Have et al., 2009). Over recent decades, there has been a general consensus that suicidal behavior is the end result of the complex interaction between many different risk factors. Whereas earlier theories focused on a single risk factor for suicidal behavior, such as entrapment (Williams 2001), escape from self (Baumeister, 1990), or a specific domain of risk such as cognition, the integrated motivational–volitional (IMV) model is one such approach that combines key factors from predominant theories into a single complex model (Figure 1: O’Connor, 2011; O’Connor & Kirtley, 2018).

The IMV model implicates many different risk and protective factors as determinants of suicide risk across three phrases. In the first phrase, the premotivational phase, the context in which suicidal thinking or behavior may emerge is described. However, within the motivational phase, suicide ideation is posited to result from feelings of defeat and entrapment, which are, in turn, moderated by feelings of thwarted belongingness and a lack of social support. The final phase, the volitional phase, is hypothesized to govern behavioral enaction, such that a suicide attempt is argued to be the result of the interaction between additional risk factors such as impulsivity or fearlessness about death and suicidal thoughts. Although different studies have confirmed the central assumptions outlined with the IMV model (see also below), these tenets have not yet to be tested within a dynamical model.

## 2 A NETWORK THEORY OF MENTAL DISORDERS

**Figure 2:**
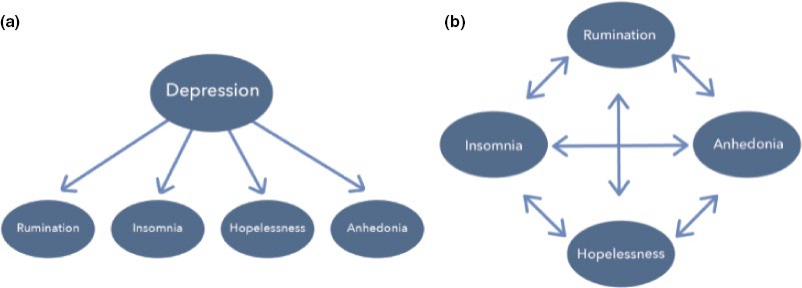
(a) Depression as a common cause of symptoms. (b) Depression emerges as a result of the interaction between symptoms [Colour figure can be viewed at wileyonlinelibrary.com]

The proposal that suicidal behavior results from the interaction of many different variables is consistent with a broader movement in psychiatry, called the network perspective (Borsboom & Cramer, 2013; Cramer et al., 2010). The central idea behind this school of thought is that a mental disorder such as major depression (MDD) is the potential consequence of symptoms that directly interact with one another in a network structure (Figure 2). That is, symptoms of MDD such as insomnia, rumination, and anhedonia do not covary because they share an underlying cause (e.g., a brain dysfunction; Borsboom, Cramer & Kalis, 2019) (see Figure 2a) but, rather, because they directly influence one another: e.g., insomnia > rumination>anhedonia (see Figure 2b). This is fundamentally different from the traditional medical model of causality, where there is a specific cause, such as a tumor, that leads to symptoms such as coughing up blood.

The application of this approach to psychopathology has proved fruitful in generating novel hypotheses and/or understanding known empirical phenomena across multiple disorders (see Robinaugh, Hoekstra, Toner, & Borsboom, 2019 for a review). When translated to the field of suicide prevention, this approach allows us to investigate the interaction between factors that are related to suicidal thoughts and suicidal behavior, rather than a latent construct called suicidality (de Beurs, 2017). In the first part of this paper, we introduce the relevant concepts within network analysis such as network density and centrality. Where possible, we refer to studies that have applied these concepts within the field of suicide prevention. As an introduction for further studies, in the Appendix S1, we offer an overview of relevant online tutorials per method.

In the second part, we move one step further toward complex system theory, which is a branch of mathematics that describes the complex behavior of systems. Such an approach sees behavior as a result of the interaction between all kinds of different processes. The understanding of network interactions and feedback loops may, for instance, help to explain how tipping points, chaos, or cycles arise. Complex system theory has been applied to understanding a wide range of phenomena, such as the weather, financial systems, biology, but also to health care and more recently to psychiatry (Bringmann & Eronen, 2018; Cramer et al., 2016; van de Leemput et al., 2014; Leemput et al., 2014; Wichers & Groot, 2016). It offers useful concepts to better model the complex interactions between variables that can result in suicidal behavior. For example, in a US study Bryan and Rudd (2018) found that change in suicidal ideation among active duty U.S. army soldiers who had a history of multiple suicide attempts was characterized by a bimodal distribution in suicide ideation. Such a distribution suggests that, for some at least, suicidal ideation alternates between stable states and tipping points (Bryan & Rudd, 2018). In the present paper, we introduce the concepts of alternative stable states, tipping points, and resilience in the context of suicidal risk, and refer to introductory papers.

## 3 PART ONE: NETWORK ANALYSIS

### 3.1 What is a network?

A typical network consists of edges and nodes. One well‐known network that comes to mind is a social network (Barabasi, 2014). In a social network, the nodes represent people or groups of people, and the edges are a quantification of their relationships, for example how many mutual friends they have. This is different from networks within the field of psychopathology. Nodes do not refer to actual physical representations, but to psychological phenomena including symptomatology that are assessed by, for example, questionnaires or clinical interviews. Whereas the edges in social networks represent actual relationships that can be counted, the edges in psychological research generally refer to the estimated statistical relationship or correlation between two nodes.

**Figure 3:**
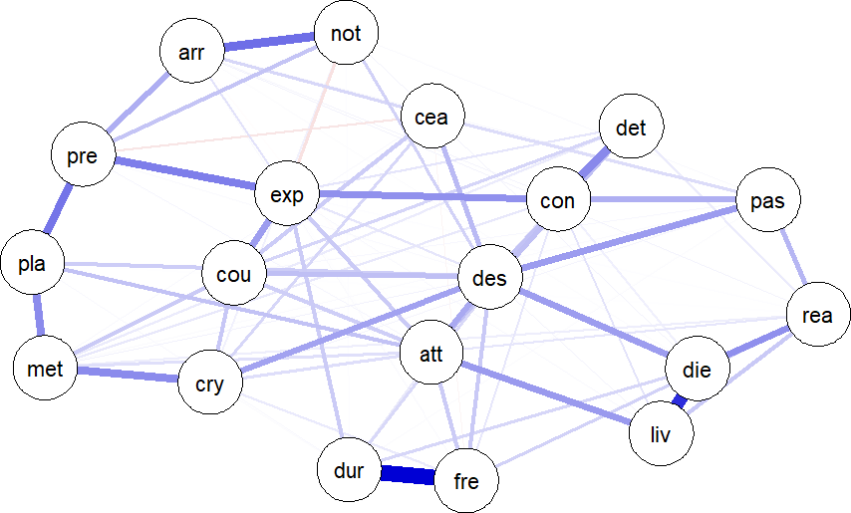
A network of the 19 separate items of the Beck scale for suicide ideation. Thicker edges present stronger associations/correlations. arr, arrangements after death; att, attitude toward suicidal behavior; cea, concealment about ideation; con, control over action; cou, courage for actual behavior; cry, cry for help versus cry for pain; des, desire to harm myself; det, deterrents of attempt; die, wish to die; dur, duration of suicide ideation; exp, expectancy of actual attempt; fre, frequency of suicide ideation; liv, wish to live; met, availability of methods; not, suicide note; pas, passive desire; pla, actual planning; pre, actual preparation; rea, reasons for living. See also de Beurs et al., 2017. See the Appendix S1 for code and data used [Colour figure can be viewed at wileyonlinelibrary.com]

An often used method to estimate networks in psychiatry is a so‐called Pairwise Markov Random Field (PMRF: Epskamp & Fried, 2016). In a PMRF, nodes are connected by undirected edges (i.e., edges with no arrowhead). When nodes are connected, they are stated to be conditionally dependent: The two nodes are related even after controlling for all other nodes in the network. An edge between two nodes can occur for several reasons. The most common scenario is a true causal relationship (e.g., entrapment ↔ suicide ideation), but the direction of the causal link cannot be inferred only from the observed relation. Alternatively, an unmeasured third node (entrapment ← cognitive reactivity → suicide ideation) could result in an edge, where cognitive reactivity is the unmeasured third node. Likewise, the absence of an edge can have several explanations, the two most common being the absence of a causal relationship, or the study has insufficient power to detect a small causal effect. Networks can include continuous items/scales, binary/categorical items, or a mixture of both. As psychological items and scales are often highly correlated, networks are usually regularized, omitting small edges (Epskamp & Fried, 2016). This means that a conservative network is estimated, resulting in the most sparse network. Most networks in the extant literature are based on cross‐sectional data. The nodes typically represent the score of a single item of a psychological questionnaire such as the Beck scale for suicide ideation, and the edges between two nodes represent the partial correlation between the two nodes, which can be either positive or negative. Figure 3 presents an example of a network of the 19 items of the Beck scale for suicide ideation (de Beurs, van Borkulo, & O’Connor, 2017). The items were answered at one moment in time by a group of 366 patients who were admitted to a hospital following a suicide attempt.

### 3.2 Network density

**Figure 4:**
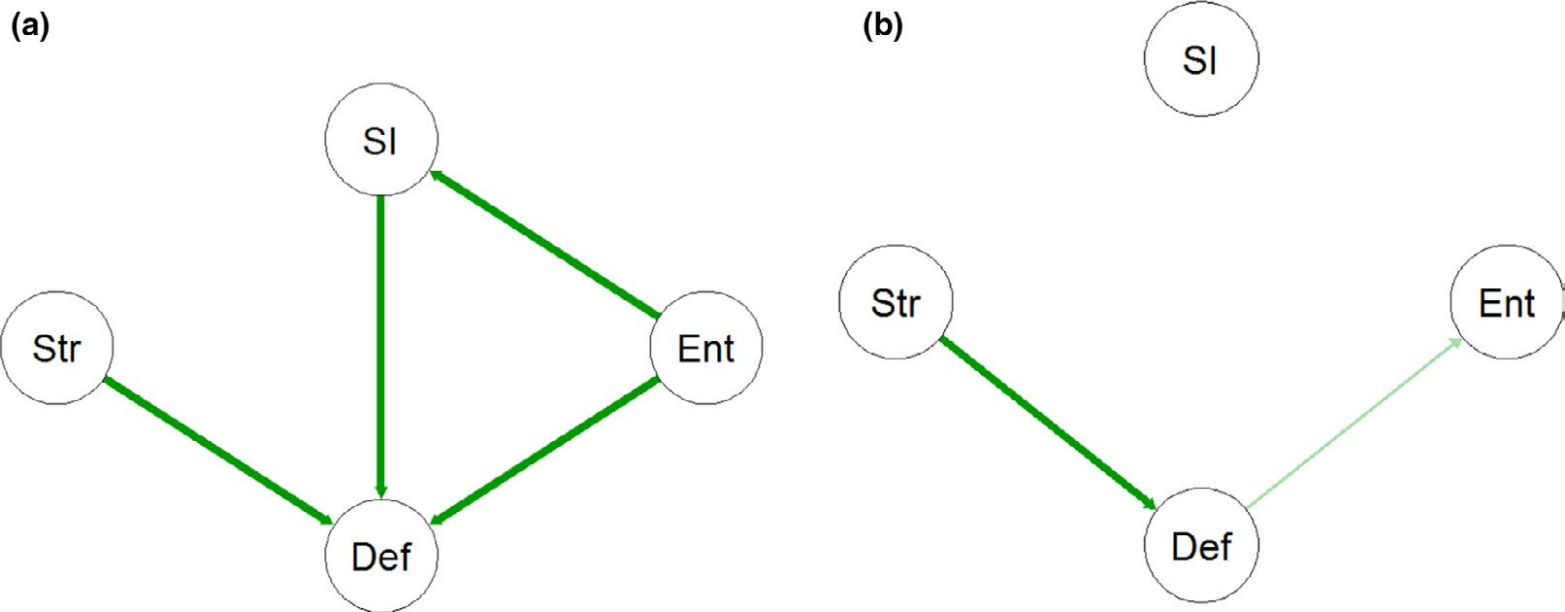
The hypothetical networks (a) and (b). Str, stress; Def, defeat; Ent, entrapment; SI, suicide ideation. A green line indicates a positive relationship between two symptoms. The thicker the line, the stronger the association. Str: stressor such as a job loss, Def: defeat, Ent: entrapment: SI: suicide ideation [Colour figure can be viewed at wileyonlinelibrary.com]

The network perspective hypothesizes that each individual has their own individual network structure (see, e.g., Cramer et al., 2016). Specifically, it is hypothesized that rather than focusing on the symptoms themselves attending to the strength of connections between symptoms can better inform us about whether someone will develop future psychopathology or not. If one assumes that edges represent positive causal interactions, an individual with a strongly connected symptom network is reasoned to be at higher risk of future psychopathology. For example, after losing their job, an individual may experience high levels of defeat that results in strong feelings of entrapment, which in turn lead to feelings of suicide ideation. This activation of symptoms can result in a reinforcing feedback loop: entrapment → suicide ideation → defeat →entrapment (Figure 4a). As is explained in part 2 of this paper, this may play an important role in the transition from being relatively low in risk to high in risk of suicidal behavior. On the other hand, someone else may feel defeated after their job loss, but defeat and entrapment are less strongly connected in their network so they feel less entrapped. Also, in the latter network, defeat and entrapment are not related to suicide ideation, meaning suicidal ideation is less likely to emerge even in the presence of high levels of defeat (Figure 4b).

At a group level, in a prospective study of psychiatric patients, those with more densely connected networks at baseline were more likely to experience another depressive episode at follow‐up compared to those with less densely connected networks (van Borkulo et al., 2015). However, this finding was not replicated in a different sample (Schweren, van Borkulo, Fried, & Goodyer, 2018). A user‐friendly method to compare the structure of groups is via the NetworkComparisontest package developed by Dr van Borkulo (Van Borkulo, Epskamp, & Milner, 2016). It uses permutation tests to investigate whether all network structures are identical (null hypotheses) or whether the null hypothesis must be rejected. A tutorial can be found online (https://cvborkulo.files.wordpress.com/2017/06/ncttutorial.pdf). A more recent package called BGGM uses Bayesian statistics to compare the structure between groups (Williams & Mulder, 2019). (https://cran.r‐project.org/web/packages/BGGM/vignettes/ppc1.html).

### 3.3 Centrality

Among the most popular metrics in network analysis are centrality estimates (Opsahl, Agneessens, & Skvoretz, 2010). There are several centrality measures, but they all relate to the inter‐connectedness of a node within a network. The most often used metric is the strength of a node, calculated by summing the size of all its edges. However, as edges can both be positive and negative, an additional *expected influence* metric has been developed, which takes negative edges into account when calculating centrality (Robinaugh, Millner, & McNally, 2016). Other popular centrality metrics are betweenness, which is the number of times a node lies on the shortest path between nodes, and closeness, which is inversely proportional to the mean shortest distance from the node to all the other nodes in the network.

**Figure 5:**
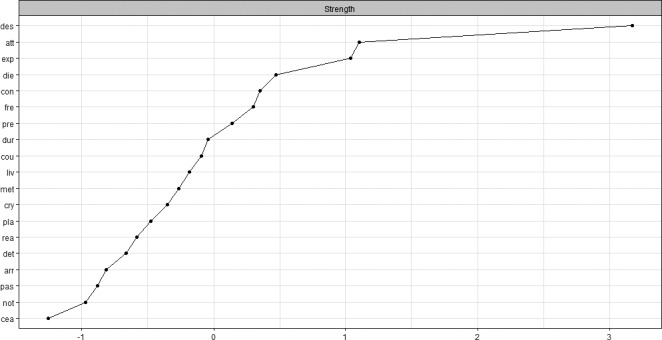
Centrality plot of the strength of each of the 19 separate items of the Beck scale for suicide ideation within the network. *X*‐axis represents standardized centrality coefficients. arr, arrangements after death; att, attitude toward suicidal behavior; cea, concealment about ideation; con, control over action; cou, courage for actual behavior; cry, cry for help versus cry for pain; des, desire to harm myself; det, deterrents of attempt; die, wish to die; dur, duration of suicide ideation; exp, expectancy of actual attempt; fre, frequency of suicide ideation; liv, wish to live; met, availability of methods; not, suicide note; pas, passive desire; pla, actual planning; pre, actual preparation; rea, reasons for living dying

As is evident in Figure 5, the item “I have a desire to harm myself” was by far the most central item from the Beck scale for suicide ideation. Initially, within network theory, the most central item was deemed the most relevant for clinical intervention. As this item has the strongest relationship with all other items, intervention on this node will most effectively influence the network. It is important to note, however, that the direction of the relationship between nodes is not clear in an undirected network (i.e., a network without arrows). Targeting a central node is only useful when the central node influences the connected symptoms, and not the other way around (Borsboom & Cramer 2013). In addition, most studies that report centrality measures are based on between participants data, and we do not know how these group centrality measures translate to the individual. The only way to validate this is to conduct experimental studies where networks before and after a manipulation of a central node are compared. However, in psychiatry, it is almost impossible to target only one node in a network, as all interventions are likely to influence other nodes as well. This prompted some authors to suggest that it may beneficial to drop the concept of centrality, which centers around a single variable and move toward the complexity of networks (Bringmann et al., 2019).

### 3.4 Network stability and accuracy

There is not yet a clear standard for calculating the required sample size to reliably estimate the strengths of the edges in a network. As a rule of thumb, one needs at least as many observations as parameters (*n* + *n* * (*n* − 1)/2 possible pairwise interactions, with n representing the number of nodes). When you have a network of 10 nodes, this translates into a minimum of 55 participants. For 20 nodes, the minimum number of participants is becomes 210, and for 30 nodes, one needs 46 participants. It has also become standard to test the stability and accuracy of a network (Epskamp et al., 2018). A link to a tutorial on how to estimate stability and accuracy is offered in the Appendix S1.

### 3.5 The utility of network analysis to test theory

Network analysis has largely been used as an exploratory tool, using variables that have been selected because of their availability rather than being driven by theory (e.g., De Beurs et al., 2017; Bringmann, Lemmens, Huibers, Borsboom, & Tuerlinckx, 2015; Fried et al., 2018). In 2019, Beurs et al. published a study with the explicit aim of understanding suicidal ideation from a network perspective by selecting variables based on psychological theory. In this study, the authors used network analysis to compare the central tenets of two different theories; the interpersonal theory of suicidal behavior (IPT: Van Orden et al., 2010) and the earlier mentioned IMV model. According to the IPT, suicide ideation emerges from the interaction of perceived burdensomeness and thwarted belongingness, whereas in the IMV model, entrapment is hypothesized to play a key role. The rationale was simple, if the data would support the IPT, then perceived burdensomeness and thwarted belongingness would be most strongly related to suicide ideation compared to any other variable. If on the other hand, the data would support the IMV model, then entrapment would be most strongly associated with suicidal ideation. When comparing the core constructs of both models, both perceived burdensomeness and internal entrapment were most strongly related to suicide ideation. Thwarted belongingness and defeat were mainly indirectly related to suicide ideation as posited by the IMV model.

The authors also estimated a network using 20 different motivational and volitional risk factors (from IMV model). Twelve of the 20 were directly related to suicide ideation after controlling for all other variables, and none of the risk factors was isolated within the network (de Beurs et al., 2019). This highlights the complex relationships between different risk factors and suicide ideation. The move from exploratory convenience networks to network analysis with a strong theoretical foundation is an important step forward in advancing our understanding. Another innovative study translated existing psychological theory and research on recurrent panic attacks into equations that explicitly define the relationships among the different symptoms in a network (Robinaugh, Haslbeck, et al., 2019). In a more theoretically oriented paper, the importance of formal theory in psychiatry was stressed, and it also offers ideas about how the network approach can inform theory (Haslbeck, Oisín, Robinaugh, Waldorp, & Borsboom, 2019).

### 3.6 Inferring network interactions from time‐series data

Although psychology is concerned with the subjective experiences of an individual, most psychological studies rely on group‐level data (Barlow & Nock, 2009). Historically, one important reason for this was that it was technically not feasible to collect large amounts of data within one person. It is only relatively recently that we have been able to conduct studies using mobile telephone technology to collect time‐series data within suicidal individuals. Initial results showed that suicide ideation fluctuates considerably over time, as do common risk factors such as hopelessness and perceived burdensomeness (Hallensleben et al., 2018; Kleiman et al., 2017). Time‐series network analysis can be conducted on a group level and on an individual level. In a recent study, ecological momentary data in depressed inpatients were collected 10 times a day, over a period of 6 days (Rath et al., 2019). In addition to suicide ideation, depressive feelings, anxiety, positive affect, perceived burdensomeness, thwarted belongingness, and hopelessness were also assessed. All variables demonstrated moment‐to‐moment variability, and substantial within person variance (Forkmann et al., 2018). Perhaps unsurprisingly, over an assessment period of about 1.5 hr, suicide ideation was mainly predicted by suicide ideation by the previous assessment of suicide ideation (at lag 1). Other risk factors did not appear to affect suicide ideation within this time span. When inspecting the network of symptoms at the same assessment (the contemporaneous network), the expected associations between, for example, hopelessness and suicide ideation were present. These results indicate that the temporal relationship between suicide ideation and risk factors may be faster than 1.5 hr, and perhaps occur nearly simultaneously.

The collection of data via a mobile phone offers novel ways of studying an individual's own dynamic network, and might be useful during treatment. An early case study in which a patient discussed their network of symptoms related to depression with a clinician indicated that it might be a feasible tool for use in clinical practice if proven to be effective (Kroeze et al., 2017). Currently, there are several ongoing studies (Stikkelbroek, Nauta, Bockting, 2019, Nuij et al., 2018) that are collecting data using mobile phones to investigate, for instance, whether an individual with a more densely connected network is indeed at higher risk of suicidal behavior and depression and anxiety compared to someone with a less dense network. Time‐series data also have the potential to detect an upcoming crisis before it takes place, as outlined below.

## 4 PART TWO: SUICIDAL BEHAVIOR AS A COMPLEX SYSTEM

One of the leading experts in network science, Professor Barabási from the Northeastern University in Boston, has stated that “networks are only the skeleton of complexity, the highways for various processes that make the world hum” (Barabasi, 2014). Complex systems are everywhere in nature, from the processes within a single cell to the climate on earth (Scheffer, 2009). A system (which is simply something concrete or abstract that is being studied) is called complex when it consists of a number of interacting elements that show some form of behavior that cannot be explained by the dynamics of the individual elements. Complex systems are nonlinear, meaning that the input in the system is not proportional to the output. For example, depression has been considered to be a complex system, since a small change in mood can have a large effect on the state someone is in. This is due to the occurrence of feedback loops in the system (e.g., low mood may lead to sleep problems or reduced social interactions, which may lead to an even worse mood). In this part of the paper, we introduce the idea that suicidal behavior can also be understood as a complex system: complex, because many clinicians and researchers argue that suicidal behavior is the end result of the interaction between many different risk factors, and cannot be explained by one single factor (Van Hemert, Kerkhof, de Keijser, & Verwey, 2012; O’Connor & Nock, 2014); and a system, because the risk factors are considered part of a system such as that proposed by the integrated motivational volitional model of suicidal behavior (O’Connor & Kirtley, 2018a).

**Figure 6:**
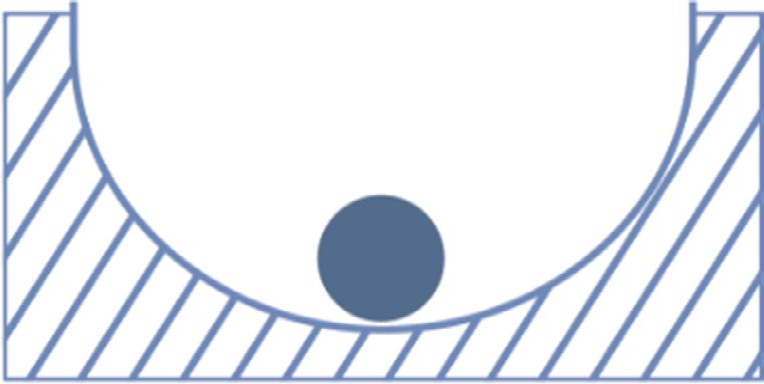
An conceptual representation of a stable state [Colour figure can be viewed at wileyonlinelibrary.com]

To understand the nonlinear dynamics of complex systems, it can be helpful to understand the behavior of dynamical systems. Dynamical systems are mathematical models that describe the time dependence of one or more variables. They can have different types of attractors, such as cycles, chaos, or stable states. A common attractor is a stable state (Figure 6). If such a system is perturbed away from the equilibrium, it eventually moves back to the stable state, due to stabilizing mechanisms. For example, one can imagine that a person's stable state is when there is no risk for suicidal behavior. Mood or other elements of a person's emotion regulation system are continuously fluctuating over time, so the ball in Figure 6 is kicked around. Still, the system is organized such that an increase in depressed feelings, for instance, because of some bad news, will be followed by a decrease in depressed feelings, for instance, because of a night's good sleep, or some physical activity.

**Figure 7:**
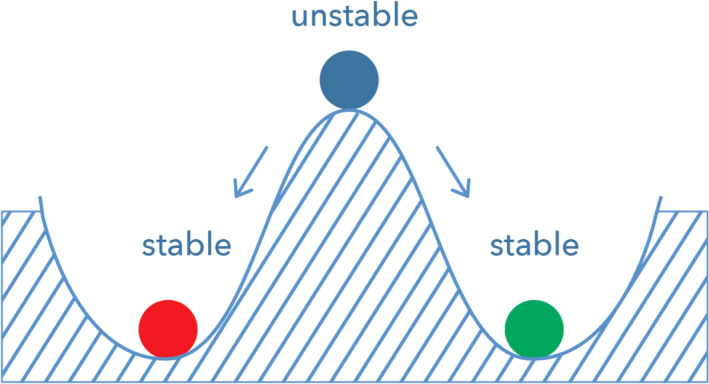
Some systems can have multiple stable states [Colour figure can be viewed at wileyonlinelibrary.com]

Some systems have alternative stable states (multiple values in the stability landscape). Alternative stable states are different stable states in which a system can be under the same conditions. Due to external stressors, or even normal fluctuations within a network over time, a system can move from one stable state to another alternative state (Figure 7). Take, for example, a population of animals that will get into trouble if their number become too low to find a mate and reproduce (Scheffer et al., 2009). A large disturbance, such as a disease, could push the population below a critical level, such that the system tips from the survival state to the extinction state, and is not able to recover. Also, if the resilience of the survival state is already low, for instance, because of anthropogenic pressure, small natural fluctuations could already trigger a tipping point to an alternative state. Translated to psychopathology, a patient could shift from a state with no risk for suicidal behavior to an alternative state with elevated risk for suicidal behavior.

**Figure 8:**
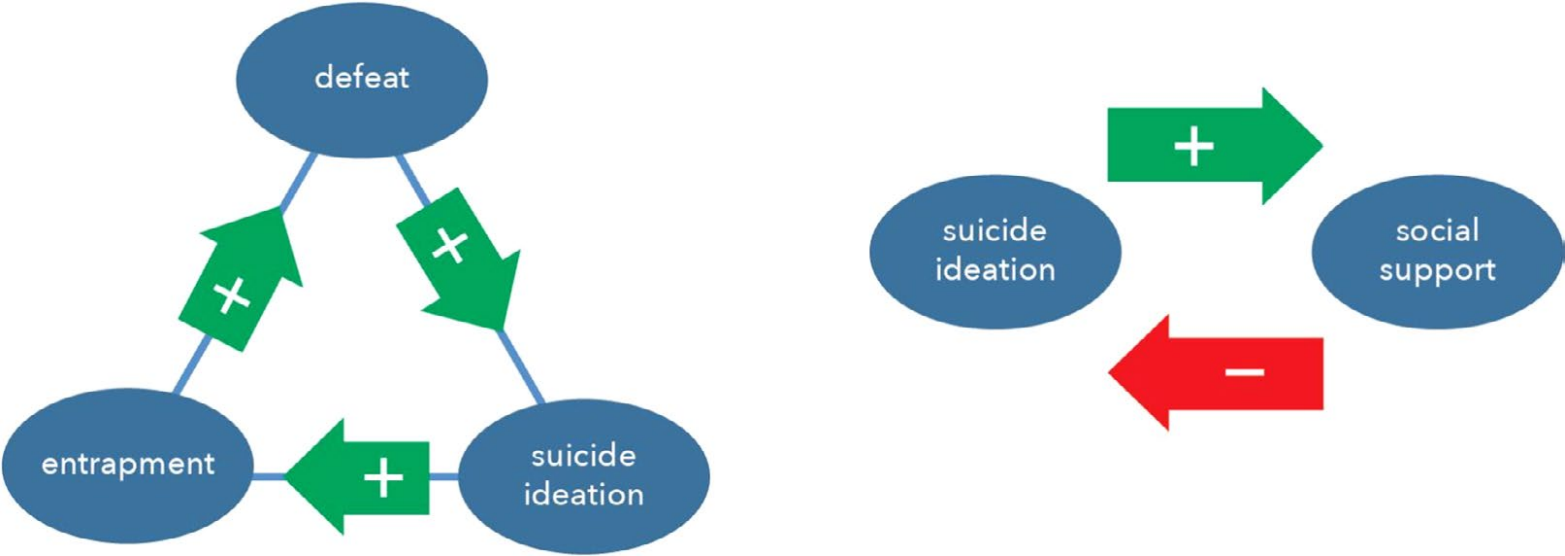
Example of a positive feedback loop (left‐hand) and a negative feedback loop (right‐hand) [Colour figure can be viewed at wileyonlinelibrary.com]

The shape of the stability landscape (e.g., Figure 7) will largely depend on the strength of positive and negative feedback loops in the system (Scheffer et al., 2009). It is important to note that the terms “positive” or “negative” are value‐free. They refer to the net sign of the overall effect of the feedback. Positive feedback loops reinforce the effect of a perturbation in the network, and thereby create an unstable intermediate state. An example of a positive feedback loop within the IMV model would be: increases in feelings of defeat → stronger feelings of entrapment → more suicide ideation → stronger feelings of defeat etc (Figure 8).

Negative feedback loops have a stabilizing effect, because they dampen a perturbation. A negative feedback loop might be: suicide ideation → more social support → less suicide ideation. When the resilience of a person erodes, stabilizing mechanisms generally weaken, while reinforcing mechanisms strengthen. As a result, it becomes easier to push a system out of its stable state, such that the valley in Figure 7 becomes more flat. Knowing when a tipping point is approaching, or in other words when resilience is decreasing, might prove to be important from an intervention perspective.

### 4.1 Cusp catastrophe model

**Figure 9:**
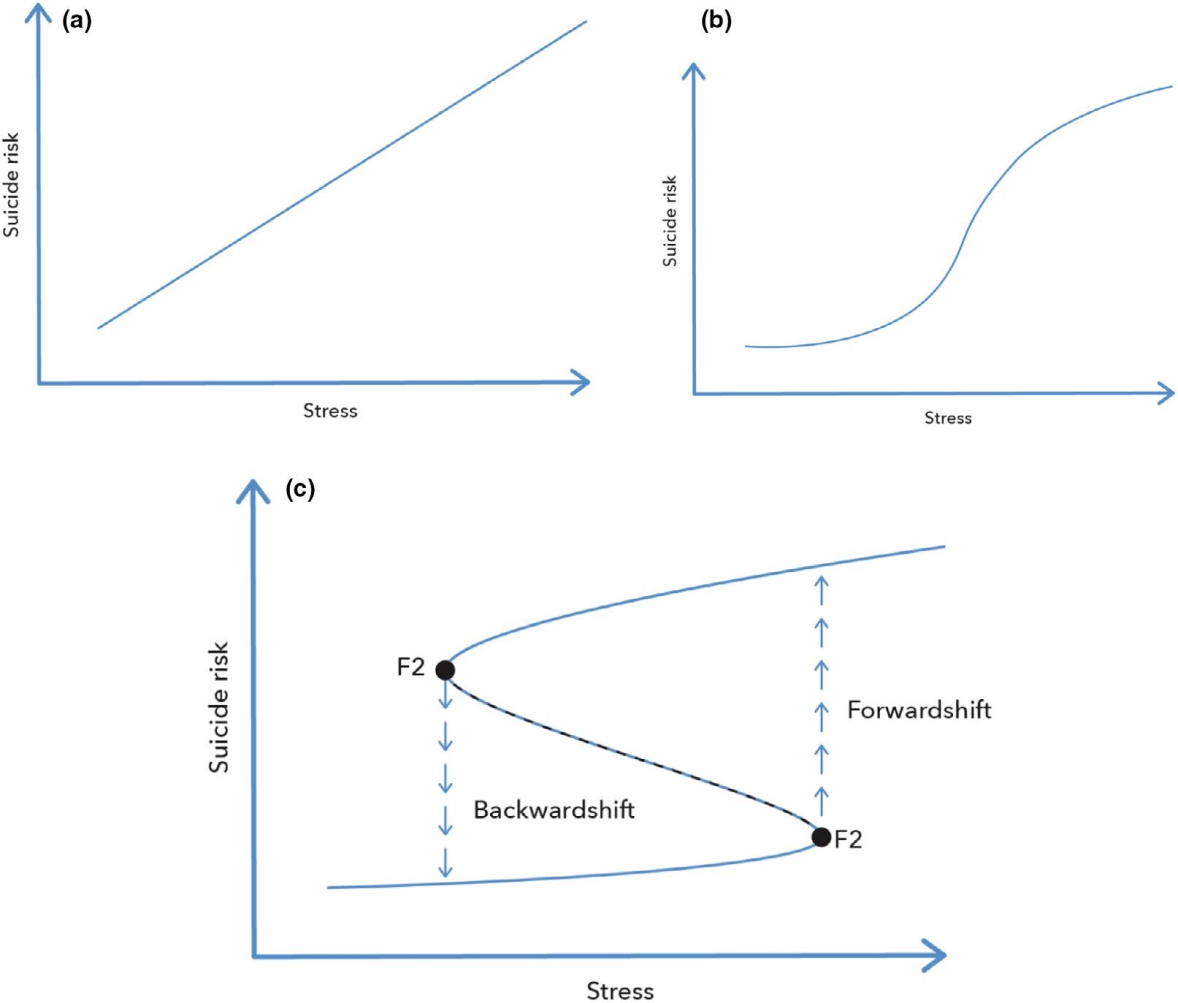
(a) Linear relationship between stress and risk of suicidal behavior. (b) Sigmoidal relation between stress and risk for suicidal behavior. (c) The relationship between stress and suicide risk as a cusp catastrophe model [Colour figure can be viewed at wileyonlinelibrary.com]

The influence of stress on complex systems or networks in systems with positive feedback loops can be understood in the context of the cusp catastrophe model (Scheffer 2009). The cusp catastrophe model is a mathematical model that can explain why relatively small changes in a parameter (in our example, small changes in stress) can result in catastrophic changes in the state of a system (in our example, a shift from the motivational to volitional phase). The main idea of the cusp model is that the stronger the positive feedback(s), the more discontinuous the network will behave under stress. In Figure 9a–c, three different kinds of hypothetical interactions between (external) stress and suicide risk are shown (Scheffer 2009). In Figure 9a, an increase in stress results in a stable increase in risk of suicide, as a patient moves in a linear way from low risk to high risk. Importantly, after stress has increased, the patient can easily go back from being at a high risk to being at a low risk when stress decreases again to a relatively normal level.

In Figure 9b, it is hypothesized that across certain ranges of stress, a patient's risk of suicide does not change very much, but when a specific stress threshold is reached, a patient will respond relatively strongly. When a positive feedback loop is really strong, it could even be that low suicidal risk and high suicidal risk represent two separate states (Figure 9c), separated by an unstable state (dashed).

**Figure 10:**
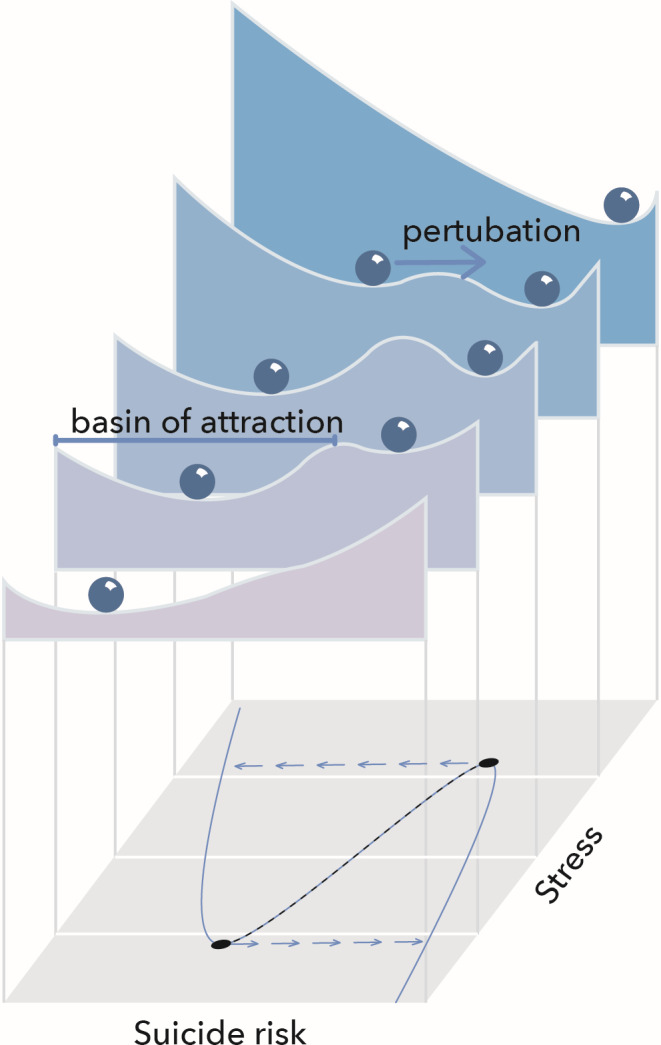
Increased stress results in loss of resilience, making the transition from one state (valley) to the other more likely. Bottom plane follows the curve of Figure 9c (Scheffer et al 2001) [Colour figure can be viewed at wileyonlinelibrary.com]

When a patient is in the lower branch of the curve (at low risk), they may become at higher risk with increasing stress, but this effect may go unnoticed. However, when a certain threshold level of stress is reached, a “catastrophic” transition from low to high risk occurs (at F1). From a clinical perspective, this is important because a very small change in stress (or any other risk parameter) would result in a very large increase in risk for suicidal behavior. When a patient suddenly becomes suicidal, we tend to look for major changes that caused the transition, but for some patients, it might be that the system was slowly getting less and less resilient, and that the sudden collapse can be explained by an already fragile system rather than a novel stressor. As visualized in Figure 10, as stress increases, the basis of attraction changes, making it more easy for the ball to reach the other stable state even after a small perturbation.

Another important aspect from a clinical perspective is that in order to get back from a high‐ to a low‐risk phase, it is not sufficient to restore stress levels to the level before the collapse. The stress level needs to go all the way back to the tipping point F2. This dependence of the current state of the system on the previous state is called hysteresis. The application of cusp catastrophe modeling within the field of suicide prevention has been elegantly demonstrated by Bryan and Rudd (2018). Within a sample of 76 active duty U.S. army soldiers, they showed that over time, those with a multiple attempt history displayed two stable states, corresponding to a high‐risk state and a low‐risk state for suicidal behavior. As depicted in Figure 9c, when a participant was in one of the states, they were more likely to stay in that state, until a new tipping point was reached. Within those soldiers who had only thought about suicide or who had a history of one suicide attempt, suicide risk tended to change in a more linear fashion, as depicted in Figure 9a. This study represents an important step forward in terms of studying nonlinear change in suicidality, especially when combined with the concept of critical slowing down (see below).

### 4.2 Critical slowing down

Importantly, there can be detectable warning signals before a system reaches a tipping point (Scheffer et al., 2009). Normally, if a person experiences, for example, a higher level of hopelessness, the level of hopelessness also goes down again after a short period of time. Indeed, recent studies using data collected via mobile phones have found that the level of well‐known risk factors such as hopelessness fluctuate heavily over time. However, when one gets near a tipping point, this fluctuation tends to slow down. If this person experiences a higher level of hopelessness compared to earlier assessments, it will take them longer to return to their normal levels. This phenomenon has been called critical slowing down (CSD: Dakos et al., 2012; Scheffer et al., 2009). A loss of resilience could be detected with perturbation experiments or by analyzing natural fluctuations around an equilibrium. If a system slows down, one could detect this as an increase in autocorrelation and variance in the time series. EMA data seem to be good candidate to investigate such changes, see, for instance, Van de Leemput et al. (2014) Wichers et al. (2016); however, these data are still rare, which makes the analysis more complicated. As summarized in the Appendix S1, many freely available tutorials exist. We do however advise to always collaborate with a methodological expert and not to use the tools as a “black box.” Also, as the field of analysis of time‐series data in human behavior is relative new, there is no preferred method of analysis for this kind of data. In an innovative study, 12 prominent EMA teams were challenged to analyze the same data from one individual patient's time‐series data. The different teams chose different analytical approaches, resulting in different outcomes (Bastiaansen et al., 2019). Many conceptual and methodological issues still need to be resolved in this field of work.

### 4.3 Critique of the network perspective

One of the main critiques reviewers often provide when evaluating the network perspective is that it is nothing more than a visualization of a correlation matrix or factor loadings. Indeed, a paper on this topic (Kruis & Maris, 2016) has shown that latent variable models and network models are statistically equivalent. So, what is the difference? First of all, network analysis offers insights into the relationships between all variables, not only into the relationships between the dependent variables and the independent variable. This provides novel insights, and it can create additional hypotheses. The visualization offers a much more intuitive way to understand data compared to a table of regression coefficients. However, it is important not to use the network graphs as modern Rorschach tests, in which the interpretation is in the eye of the beholder. The nodes in a network should be interpreted with caution as they depend on the software settings. The visualization is merely a useful tool to derive new hypotheses, it should not be used as a confirmatory tool. As stated in the second part of this paper, conceptualizing psychopathology as a network of symptoms offers a new set of tools derived from complexity theory that may help us better understand complex dynamic phenomena such as psychiatric illness and suicide risk.

It is also important to highlight that even when different scientific models have similar statistical properties, this does not necessarily mean that they are theoretically similar (https://psych‐networks.com/meaning‐model‐equivalence‐network‐models‐latent‐variables‐theoretical‐space/). Network theory offers a completely different way of thinking about psychopathology when compared to latent modeling. In the latent model, making a change to a single symptom would not directly affect the other symptoms. However, from a network perspective, changing one variable may have consequences for the whole network, at the very least for the nodes directly related to the targeted node.

Many technical challenges remain. As noted above, when applying networks, the state of the science is to estimate regulated networks using LASSO regularization. However, it has been found that under certain circumstances, the specificity is lower than expected (Williams & Rast, 2019). This insight has resulted in an update in the qgraph package, offering novel ways to estimate a network. Another technical critique relates to the replicability of networks. Networks require large sample sizes to be stable, and many researchers do not check the robustness of the network. The limited replicability has also been the topic of a recent debate (Borsboom et al., 2017; Forbes, Wright, Markon, & Krueger, 2017a, 2017b). Another challenge is that when a factor is stable, it cannot correlate strongly with other variables. For example, if we study risk factors within a highly suicidal group of patients, it might be that suicide ideation will not be connected strongly to other nodes within the network, because all participants will score high on suicide ideation. The variance of suicide ideation is then much smaller compared to the variance of other items, resulting in small connections with the other items (Terluin, De Boer, & De Vet, 2016). Although beyond the scope of this paper, it is important to acknowledge that there are measurement issues around the assessment of psychological factors and associated scales more widely. Often, scales are not well validated, or they seem to measure something different from what they purport to measure. There are at least 280 scales to measure depression, and sometimes different scales lead to different outcomes. The variety of scales used and the limited validity of the scales make replication of results difficult (Flake & Fried, 2019).

Another critique is that most network studies are based on cross‐sectional data, and provide insight into static relations between symptoms at a group level. Cross‐sectional networks do not allow us to study the individual dynamic interactions between symptoms, as a result, time‐series analyses are therefore a logical way forward. However, time‐series analyses within the field of network analysis is in its early stage, leaving open fundamental questions about how best to estimate a network over time.

### 4.4 Future directions

One of the key questions within suicide prevention is why a minority of people eventually act on their suicidal thoughts, while the overwhelming majority do not. Future studies using ecological momentary data such as from the CASPAR study offer the opportunity to test whether an individual with a strongly connected network of risk factors for suicidal behavior is indeed more at risk of suicidal behavior over time when compared to an individual with a more weakly connected network. These data may also allow us to come to formal and quantified theories, as proposed by Haslbeck et al. (2019). Formal theories are needed to really improve our understanding of mental disorders, and to be able to provide better treatment. These formal theories then inform novel data collection, yielding findings that can be used to improve the initial formal theory.

Although we have focused on networks of psychological symptoms, complexity theory applies to all types of information including genetic, metabolic, social, and environmental data. The field of suicide research and prevention should aim to gather such information routinely as we move forward in the decades to come. To this end, the University of Amsterdam has established a multidisciplinary research institute that focuses on all of the different levels of influence on mental health, ranging from the genes to the urban living environment (https://www.uva.nl/en/shared‐content/zwaartepunten/en/urban‐mental‐health/urban‐mental‐health.html). The future of suicide prevention is interdisciplinary, with geneticists, experimental psychologists, applied psychologists, psychiatrists, people with lived experience, ecologists, computer scientists, policymakers, sociologists, and colleagues from other disciplines all working together to advance our understanding of the complexity of suicidal behavior.

## 5 CONCLUSION

Clinicians and scientists more and more conceptualize suicidal behavior as a result of the complex interaction between many different variables. Novel statistical techniques such as network analysis can help us to better study this complexity. Network analysis can be seen as a starting point to move from traditional linear thinking toward a dynamical model of a complex system. Only recently have researchers started in earnest to apply concepts from complex systems thinking to the field of psychopathology and suicidology. Such a collaborative approach offers exiting and promising possibilities for our understanding of suicidal behavior.

## Supporting information

Supplementary Material
