# Supplementary material for: A network perspective on suicidal behavior: Understanding suicidality as a complex system
Source: Suicide Life Threat Behav. 2021 Feb 24;51(1):115–26. doi: 10.1111/sltb.12676 (PMC7986393; doi:10.1111/sltb.12676)
Supplement: Supplementary file 1 — Supplementary Material [file SLTB-51-115-s001.docx]

Supplementary file

Network analysis

Several tutorials and workshops are available to help a researcher apply network analysis to their own data. A good place to start is the freely available material from Professor Denny Borsboom’s group at the University of Amsterdam (<https://osf.io/fv24x/>). The material also includes introductory videos to install R and Rstudio. A tutorial using data from suicidal patients was developed for a presentation at the 30^th^ World Congress of the International Association for Suicide Prevention (IASP). This demonstrates how to estimate networks using the R packages bootnet (Epskamp, Borsboom, Eiko, & Fried, n.d.; Epskamp & Fried, 2017) and qgraph (Epskamp, Cramer, Waldrop, Schmittmann, & Borsboom, 2012) and is available online (<https://derekdebeurs.github.io/IASP2019/>).

As the data, the software and the code are freely available, the tutorial offers an initial step to start estimating networks using one’s own data. Collaboration with a network analysis expert is recommended, and it is important not to use the tools blindly as ‘black boxes’. Below, we highlight some of the tutorials that address the topics discussed in the paper.

Inferring network interactions from symptom correlations

In R, a network can be easily visualized using the qgraph package(Epskamp et al., 2012). First, one needs to install all of the required packages for this supplementary file in R. This only needs to be done once:

list.of.packages <- c("bootnet", "qgraph", "RCurl", “MGM” )

new.packages <- list.of.packages[!(list.of.packages %in% installed.packages()[,"Package"])]

if(length(new.packages)) install.packages(new.packages)

Then, one can estimate a network by first creating a simple matrix of three rows and three columns, and then feed that matrix in the qgraph package:

library(qgraph) ## One always needs to load the packages into a new R session

example <- matrix ( c(0,4,3,

4,0,0,

3,0,0), nrow=3,

ncol=3)

colnames(example) <- c("Cog","Ent", "SI")

qgraph(example, layout = "circle", vsize = 15)


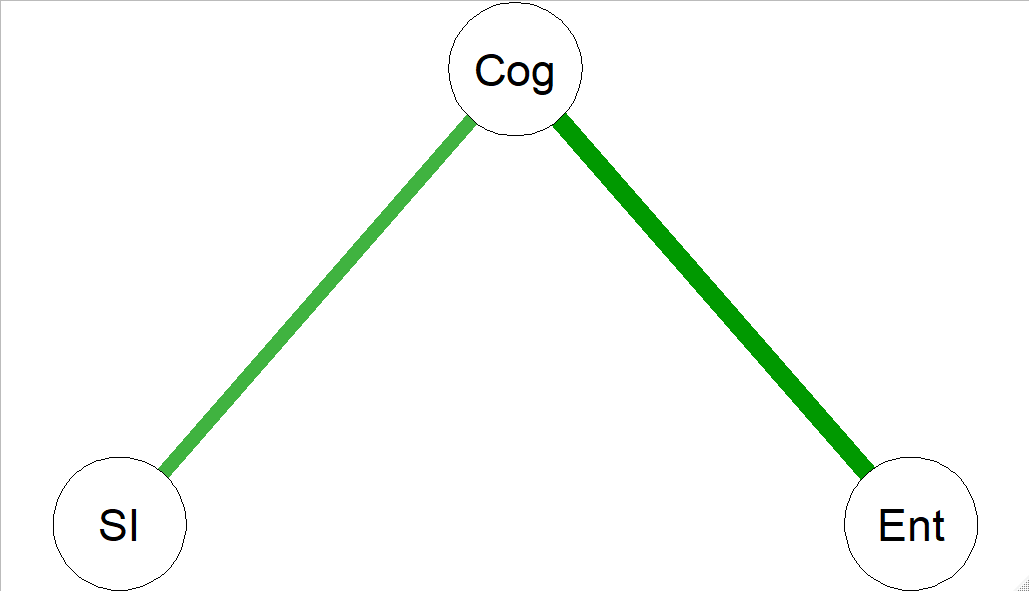


An example of a matrix visualization in R, using the qgraph package. Edges represent partial correlations between the nodes.

Network analysis of cross-sectional data. Rebuilding figure 3 of the article.

Below, the R code and data are presented from a paper that estimated the network structure of separate items of the Beck Scale for Suicide Ideation (de Beurs, van Borkulo, & O’Connor, 2017). A partial correlated network with a LASSO regulation is estimated for the 19 separate items of the Beck Scale for Suicide Ideation. The full tutorial can be found online: <https://derekdebeurs.shinyapps.io/suicidenetwork/#section-introduction>.

library(RCurl) ## package to download data from websites

library("bootnet") ## package to estimate networks

x <-getURL("https://raw.githubusercontent.com/derekdebeurs/SLBT2020/master/data_slbt.csv")

data <- read.csv(text = x)

data <- data[,-1]

Network1 <-estimateNetwork(data, default = "EBICglasso")

plot(Network1, layout = 'spring', cut = 0)

Centrality

The centrality of the network can be estimated using the following line of code:

centralityPlot(Network1, orderBy = "Strength")

Network stability and accuracy:

A tutorial on network stability and accuracy can be found via <https://psych-networks.com/r-tutorial-power-issues-robustness-network-models/>.

Cusp catastrophe models:

Simulation studies have shown how the cusp catastrophe model can be used as a method to study depression (Cramer et al., 2016). Individuals with more densely connected networks, and therefore stronger feedback loops, were more likely to be ‘pushed into’ a depressed state when exposed to stress, and indeed they showed hysteresis (Cramer et al., 2016). We refer the reader to an online simulation that allows one to explore different parameters (<http://simulations.psychosystems.org/>).

Alternative stable states

Scheffer & Carpenter (2003) have written a useful paper that reviews all of methods to detect alternative stable states.

Early warning signals

A paper that describes different methods to detect early warning signals is available (Dakos et al., 2012) and a R package developed by the first author is also available: <https://github.com/earlywarningtoolbox/earlywarnings-R>
